# Association Between Language Use and ICU Transfer and Serious Adverse Events in Hospitalized Pediatric Patients Who Experience Rapid Response Activation

**DOI:** 10.3389/fped.2022.872060

**Published:** 2022-07-05

**Authors:** Jessica E. McDade, Aleksandra E. Olszewski, Pingping Qu, Jessica Ramos, Shaquita Bell, Alicia Adiele, Joan Roberts, Tumaini R. Coker

**Affiliations:** ^1^Division of Critical Care, Department of Pediatrics, University of Washington and Seattle Children's Hospital, Seattle, WA, United States; ^2^Division of Critical Care, Department of Pediatrics, McGaw Medical Center of Northwestern University, Chicago, IL, United States; ^3^Ann and Robert H. Lurie Children's Hospital of Chicago, Chicago, IL, United States; ^4^Seattle Children's Research Institute, Seattle Children's Hospital, Seattle, WA, United States; ^5^Center for Diversity and Health Equity, Seattle Children's Hospital, Seattle, WA, United States

**Keywords:** disparities, hospital medicine, critical care, language interpretation, interpreter use

## Abstract

**Background:**

Hospitalized patients and caregivers who use a language other than English have worse health outcomes, including longer length of stay, more frequent readmissions, and increased rates of in-hospital adverse events. Children who experience clinical deterioration (as measured by a Rapid Response Team event) during a hospitalization are at increased risk for adverse events and mortality.

**Methods:**

We describe the results of a retrospective cohort study using hospital records at a free-standing, quaternary children's hospital, to examine the association of language of care with outcomes (transfer to intensive care, adverse event, mortality prior to discharge) following Rapid Response Team event, and whether increased interpreter use among patients who use a language other than English is associated with improved outcomes following Rapid Response Team event.

**Results:**

In adjusted models, Rapid Response Team events for patients who use a language other than English were associated with higher transfer rates to intensive care (RR 1.1, 95% CI 1.01, 1.21), but not with adverse event or mortality. Among patients who use a language other than English, use of 1-2 interpreted sessions per day was associated with lower transfer rates to intensive care compared to use of less than one interpreted session per day (RR 0.79, 95% 0.66, 0.95).

**Conclusion:**

Rapid Response Team events for hospitalized children of families who use a language other than English are more often followed by transfer to intensive care, compared with Rapid Response Team events for children of families who use English. Improved communication with increased interpreter use for hospitalized children who use a language other than English may lead to improvements in Rapid Response Team outcomes.

## Background

Several studies have shown that children of families with limited English language proficiency, or those who use a language other than English (LOE) for medical care, have worse health outcomes, (1, 2) receive lower quality care, (2, 3) and report worse patient experiences of care (4–6) compared with children of English-speaking families. Among hospitalized children, studies have shown increased serious adverse events, (7) and increased likelihood to transfer to the intensive care unit within 24 hours of admission, (8) for children of families who use a LOE compared with English-speaking children and families. Language spoken does not have a biological mechanism to explain these disparities, suggesting that inequitable provision of care may contribute. In fact, differences in treatment have been demonstrated in pediatric populations; children of families who use LOE, compared with English-speaking children, receive different treatment, including worse post-operative pain management, (3) and increased testing such as chest x-rays and blood tests for children with bronchiolitis (2).

The reasons for these disparities in care and outcomes are likely multifactorial, with pre-hospital health and in-hospital social determinants of health both playing important roles (9). The factors contributing to these disparities in treatment during a hospitalization are also multifactorial, with evidence showing that barriers to effective communication between the healthcare team and family may also play a role. Among patients who use LOE, increased rates of interpretation use by provider teams are associated with improved outcomes, improved quality of care, and better patient satisfaction among adult and pediatric patients (5, 8, 10, 11). Despite this, interpretation is underutilized, with rates reported at 39–45% of conversations with patients and families who use LOE (8, 12).

Effective communication between a caregiver and provider team may be particularly salient for hospitalized children who require activation of a rapid response system, as these represent patients with a higher likelihood of clinical deterioration. During these events in a hospital stay, often referred to as Rapid Response Team events, or “RRT”, children are evaluated with a pediatric intensive care unit (ICU) team for possible admission to the ICU. Our study objective was to determine whether outcomes for hospitalized pediatric patients who experienced an RRT were associated with patient language preference. We evaluated three primary outcomes: rate of transfer to the ICU following an RRT, rate of serious adverse event following an RRT, and mortality prior to discharge. We hypothesized that among patients who experienced an RRT, children of families who use LOE (henceforth, “patients who use LOE”) are associated with worse outcomes, and that for patients who use LOE, frequency of interpreter use would mitigate these disparities.

## Methods

### Study Design and Sample

We conducted a retrospective single-site cohort study of all pediatric patients hospitalized in an urban, pediatric tertiary institution's acute care wards including medical, rehabilitation, cancer and surgical units, between October 2016 and October 2019 who experienced at least one RRT. In our institution, an RRT can be called by any member of the medical team, including a patient's caregiver. There are no automatic or scoring system-based ways for an RRT to be called. The RRT team includes an ICU nurse, an ICU provider, a respiratory therapist, and the patient's floor medical team. Patients were excluded if they were admitted to the inpatient psychiatry facility or if there was missing data for our measured outcome variables (1.9% of all patients). The Seattle Children's Institutional Review Board approved this study.

### Outcomes

We examined three binary outcome variables: (1) transferring to the ICU following an RRT, (2) having a serious adverse event following an RRT, and (3) mortality prior to discharge. The first two outcomes were measured at the level of the RRT event (more than one RRT per hospitalization encounter was possible) whereas we measured mortality at the hospitalization encounter level (each hospitalization could have a single mortality outcome). Transfer to the ICU was defined by the date and time stamp in the electronic medical record between acute care and an ICU of <4 h following activation of the index RRT. For the outcome of rate of serious adverse event following an RRT, we used chart data on the number of “rescue events”, defined as the requirement of new non-invasive or invasive positive pressure ventilatory support, or initiation of inotropic support within 2 h of ICU transfer. Patients who experience a rescue event have higher mortality, and this was used as a proxy for measurement of other types of adverse events (13). For the rest of the text, we will use the term “adverse event” to mean an event that met any of those criteria.

### Exposure

The primary exposure variable was a binary variable, and measured whether, on admission, parents identified English as their language for communication (and thus were considered to be English-speaking) or identified a language other than English as their language for communication (and thus were considered to use LOE).

### Covariates

Covariates anticipated to confound the relationship between language use and transfer to the ICU, adverse event, or mortality were considered when designing our multivariable model. The Pediatric Medical Complexity Algorithm (PMCA) (14) is a publicly available algorithm for identifying children with medical complexity, as these patients are at higher risk of mortality (15, 16). In our analysis we categorized patients based on PMCA: no chronic disease, non-complex chronic disease (NC-CD), or complex chronic disease (C-CD), as previous studies have done (15–17). Patient public insurance status has been shown to be associated with higher all-cause in-hospital mortality, (18) and in our study was measured as a binary variable (private insurance vs. public or no insurance (0.25% of encounters were for uninsured patients). A patient's experience of racism from provider teams has been shown to impact outcomes in several clinical domains, (19–21) and thus could also impact outcomes following an RRT, so we examined the potential to include race/ethnicity as a covariate in our models as well.

### Statistical Analysis

Descriptive statistics were used to characterize the demographics of the overall study population, as well as of the two study populations. Data are presented as frequencies and percentages for categorical variables and medians and interquartile ranges (IQR) for numeric variables.

For the RRT level outcomes (whether a patient transferred to ICU following an RRT and whether a patient had an adverse event following an RRT), a preliminary fitting of the data with random effect models showed that patient random effects contributed <4% of the total variation in these outcomes and suggested including just the hospitalization random effects would be sufficient. On the other hand for the hospitalization-level outcome “survival to discharge,” the data suggested random effects at patient level would be needed. However, fitting mixed models on the data led to non-convergence in some situations. Therefore, we instead employed generalized estimating equations (GEE) to analyze the data while leveraging the knowledge about correlations from our preliminary random effect model fitting. Specifically, we used GEE Poisson regression models to analyze (1) the RRT-level outcomes assuming exchangeable correlations within hospitalizations and (2) the hospitalization-level outcomes assuming exchangeable correlations within patients. Adjusted models controlled for PMCA category and insurance category.

To understand whether frequency of interpreter use by providers caring for the patients who use LOE in the study population was associated with differences in our outcome variables among this sub-population, we conducted an analysis limited only to hospitalizations for patients who use LOE. A measure of interpretation rate was calculated by counting all interpreted sessions within one patient's hospitalization (phone, video, and in-person), and dividing that by the length of hospital stay in days. This was then categorized into a binary variable, with a cutoff of 1 interpreted session per day of hospital stay (≤1 vs. > 1).

To model the incidence rate ratio (IRR) of each outcome among patients who use LOE who experienced different categories of daily interpretation, we again used Poisson regression models with GEE method to account for correlations due to multiple RRTs during the same hospitalization for the RRT-level outcomes, or multiple hospitalizations for the same patient for the hospitalization-level outcome.

## Results

### Characteristics of Study Population

A total of 2,040 unique hospitalizations with at least one RRT, between October 2016 and October 2019 were included in this study. Of these hospitalizations, there were 1,730 unique patients, and 2,823 unique RRTs. The top diagnoses represented by the study subjects are in Supplemental Table 1. The majority of hospitalizations were for English-speaking patients (*n* = 1,751; 86%), and complex chronic patients (*n* = 1,532; 75%). The proportion of complex chronic patients was higher among patients who used LOE (83% for those who used LOE vs. 74% for English-speaking patients, *p* = 0.003; Table 1). More patients who used LOE had either public or no insurance compared to English-speaking patients (85% compared to 49%, *p* < 0.001).

**Table 1:** Demographics of 2,040 hospitalizations during the study period.

**Variable**	**Level**	**Overall**	**English-speaking patients**	**Patients who use LOE**	***P*-value**
All hospitalizations		2,040	1,751	289	
Patient Race/Ethnicity					<0.001
	White	860 (42%)	843 (48%)	17 (6%)	
	Black or African American	150 (7%)	129 (7%)	21 (7%)	
	Latino or Latina	460 (23%)	284 (16%)	176 (61%)	
	Asian	160 (8%)	115 (7%)	45 (16%)	
	Native American, Alaskan Native, or Native Hawaiian	87 (4%)	84 (5%)	3 (1%)	
	Two or more races	114 (6%)	113 (6%)	1 (<1%)	
	Other race	99 (5%)	84 (5%)	15 (5%)	
Insurance Type					<0.001
	Private	926 (46%)	892 (51%)	34 (12%)	
	Public or Uninsured	1,103 (54%)	856 (49%)	247 (85%)	
PMCA					0.003
	Non-chronic	225 (11%)	204 (12%)	21 (7%)	
	Non-complex chronic	283 (14%)	255 (15%)	28 (10%)	
	Complex chronic	1,532 (75%)	1,292 (74%)	240 (83%)	

*Data are shown as n (%)*.

More than half (54%) of hospitalizations were for patients who had public or no insurance, and 42% of hospitalizations were for patients who self-identified as White race/ethnicity, 7% for patients who identified as Black or African American, 23% as Latinx, 8% Asian, 4% Native American, Alaskan Native, or Native Hawaiian, and 11% other race or two or more races (Table 1). The majority of hospitalizations were for patients who survived to discharge (1,954; 96%). Adjusted models controlled for PMCA category and insurance category. Our adjusted models did not include race/ethnicity, because we found a very small number of patients who use LOE in some race/ethnicity categories.

### Association Between Language Use and Outcome Measures

Among the 2,040 hospitalizations, 16 hospitalizations and 100 RRTs were missing either the exposure variable, a covariate, or an outcome measure, and were excluded from analysis, leaving 2,783 RRTs from 2,024 hospitalizations and 1,717 patients. The distributions of each outcome across the English-speaking group and the group using LOE are shown in Table 2. In adjusted models, an RRT for a patient who used LOE was more likely to result in a transfer to the ICU than RRTs for English-speaking patients (IRR 1.10, 95% CI 1.01, 1.21; Table 3). There was no association between language use and experiencing an adverse event, or death prior to discharge (Table 3). Our study was powered to detect a 7.5% difference in transfer rates, a 4.2% difference in adverse event rate, and a 3.7% difference in mortality (22).

**Table 2:** RRT-level summary of each of the three RRT-level measured outcomes, for 2,783 included RRTs.

**Variable**	**Overall (*n* = 2,783)**	**English-speaking patients (*n* = 2,371)**	**Patients who use LOE (*n* = 412)**	***P*-value**
Survival to discharge	2,648 (95%)	2,260 (95%)	388 (94%)	0.383
Adverse event following the RRT	205 (7%)	168 (7%)	37 (9%)	0.209
Transfer to ICU following the RRT	1,482 (53%)	1,245 (53%)	237 (58%)	0.067

**Table 3:** Results of adjusted and unadjusted Poisson regression models associating language use with each measured outcome.

**Outcome**	**Unadjusted**	**Adjusted**
**Measure**	**IRR (95% CI)**	**IRR (95% CI)**
Survival to discharge^a^	0.99 (0.96, 1.01)	0.99 (0.96, 1.02)
Adverse event following the RRT	1.28 (0.9, 1.81)	1.16 (0.8, 1.69)
Transfer to ICU following the RRT	1.11 (1.02, 1.21)	1.1 (1.01, 1.21)

a *Survival to discharge was measured at the hospitalization-level using hospitalization-level covariates, with all other outcome variables measured and analyzed at the RRT-level*.

### Secondary Analysis: Association Between Daily Interpreter Use Frequency and Outcome Measures

Among hospitalizations for patients who use LOE, the median number of interpreted sessions per day was 1.5 (IQR 0.6, 2.7), which included in-person, video, and phone interpreted sessions. Our three outcome measures for patients who use LOE, stratified by number of interpreted sessions per day of hospital stay, can be found in Table 4. In adjusted models, >1 interpreted session per day was associated with reduced rates of ICU transfer (IRR 0.83, 95% CI 0.73, 0.95; Table 5), compared with ≤ 1 interpreted sessions per day. Rate of interpreter use was not associated with adverse event rate or mortality prior to discharge in either model (Table 5).

**Table 4:** Outcome measures stratified by the category of interpreter use per day of hospital stay during 412 RRTs and 281 hospitalizations from 229 patients who use LOE in the study population.

**Outcome**	**Adjusted IRR (95% CI)**	
	**≤1 interpreted**	**>1 interpreted**
	**sessions per**	**sessions per**
	**day (*N* = 144)**	**day (*N* = 268)**
Survival to discharge^a^	140 (97%)	248 (93%)
Adverse event following the RRT	17 (12%)	20 (7%)
Transfer to ICU following the RRT	90 (63%)	147 (55%)

a *Survival to discharge was measured at the hospitalization-level using hospitalization-level covariates, with all other outcome variables measured and analyzed at the RRT-level*.

**Table 5:** Results of adjusted Poisson regression models associating number of interpreted sessions per day (as a binary variable) of hospital stay with each of our measured outcomes during 412 RRTs and 281 hospitalizations from 229 patients who use LOE in the study population.

**Outcome**	**Adjusted IRR (95% CI)**	
	**≤1 interpreted**	**>1 interpreted**
	**sessions per**	**sessions per**
	**day (*N* = 144)**	**day (*N* = 268)**
Survival to discharge^a^	(ref)	0.97 (0.92, 1.03)
Adverse event following the RRT	(ref)	0.7 (0.38, 1.28)
Transfer to ICU following the RRT	(ref)	0.83 (0.73, 0.95)

a *Survival to discharge was measured at the hospitalization-level using hospitalization-level covariates, with all other outcome variables measured and analyzed at the RRT-level*.

## Discussion

In this retrospective study of hospitalizations in a tertiary pediatric hospital, RRTs for patients who use LOE were more likely to be followed by a transfer to the ICU than RRTs for English-speaking patients. RRTs for patients who use LOE were not more likely to result in adverse event, and hospitalizations for patients who use LOE who experienced an RRT were not associated with higher mortality prior to discharge. Among patients who use LOE who experienced an RRT, rates of interpretation use were low. RRTs among patients who use LOE with lower rates of interpretation were more likely to result in ICU transfer. Our findings add to existing literature showing differences in outcomes for children who use LOE compared to English-speaking children, and our findings raise concern for communication barriers contributing to these disparities (1–3, 8).

Our finding that RRTs for patients who use LOE were more likely to be followed by a transfer to the ICU is consistent with previous work that shows pediatric patients who use LOE in the ED are more likely to transfer to the ICU within 24 h of admission to the hospital (8). The goal of RRTs is to recognize clinical change early enough to act and improve outcomes, and we did not find any disparities in adverse events or deaths after ICU transfer. However, our findings suggest either that at time of RRT call, patients who use LOE are sicker and more likely to require ICU transfer than English-speaking patients, or that for similar clinical presentations, providers are more likely to choose to transfer patients who use LOE to the ICU compared to English-speaking patients. Potential inadequate communication during and preceding an RRT for patients who use LOE compared to English-speaking patients may contribute to differential decision for ICU transfer. Supporting this, interpreter use in our study population who use LOE was low, and there was an association with higher rates of transfer to the ICU among hospitalizations for patients who had less than one interpreted session per day compared to those who had more than one per day.

If at the time of an RRT, patients who use LOE are more likely to be more acutely and severely ill compared to English-speaking patients, our findings would support the hypothesis that RRTs are called later in an illness course for patients who use LOE compared to English-speaking patients. This supports a hypothesis that signs of illness may have been missed and appropriate care may not have been instituted as early in the illness courses of patients who use LOE. Further, if patients who use LOE are sicker at the time of RRT call, this may be influenced by inadequate communication with families who use LOE, such that medical teams are less aware of clinical changes at early stages. Previous work has also posited that under-triage for patients who use LOE may be due to poor communication with families about symptoms due to the language barrier, (7) and that for patients who are triaged at lower acuity levels, interpretation is less likely to be used (8). Differential transfer to the ICU may also be due to inadequate communication influencing team decision-making at the time of RRT call, which is supported by growing evidence among pediatric populations that provider teams make different clinical decisions for patients who use LOE compared to English-speaking patients (2, 3, 7, 8, 23, 24).

These findings suggest that interventions aimed at increasing interpretation use may improve patient outcomes. Future work should explore reasons for low interpretation use, and factors associated with increased interpreter use, to inform interventions. Potential interventions include increasing access to interpretation resources, (5, 11) process changes to identify patients who may benefit from interpretation and communicate interpretation need and use, (8, 12) education of providers and nurses, (1) standardized and protocolized processes for clinical team communications during RRTs, and changes to clinical workflow such as adjusting nursing assignments and rounding schedules to make use of interpretation less of a barrier. It is important that future work includes perspectives of families who use LOE, with the ultimate goal to address their communication needs and barriers.

### Limitations

Results of this single center study may not be generalizable to all other hospital settings, and the retrospective design limits ability to evaluate causation or mechanism. Most patients who use LOE in this study identified Spanish as their language for care, limiting generalizability for patients who use other languages. Because of our sample size, and because most families who use LOE identified as one race (Latino/Latina), we were unable to use race/ethnicity in our model as a proxy for experiencing racism. Our sample was limited to hospitalizations with an RRT, which allowed us to examine differential outcomes of RRT, but limited our ability to understand whether and when an RRT is utilized for patients who use LOE compared to English-speaking patients. There is potential for under-counting of interpreted sessions per day, as we were unable to include instances where a bilingual provider cared for a family using LOE. Further, we only have data on number of interpreted sessions per day, and thus were not able to evaluate outcomes with respect to length or content of sessions, or factors related to family communication preferences or presence at the beside. Our outcomes focused on mortality, ICU transfer, and adverse event rate; we did not consider other outcomes, such as hospitalization cost or family mental health. Additionally, our model could be impacted by unmeasured confounding, specifically for severity of illness at time of RRT or for ICU capacity (both of which we were unable to include due to lack of data availability). Many factors during a hospitalization may affect adverse events and mortality, limiting our ability to draw conclusions about the lack of differences in these outcomes for our two study populations.

## Conclusions

We found that the decision to transfer a patient to the ICU following an acute evaluation for deterioration on the floor (via RRT) varied by patient language use, with RRTs for patients who use LOE more likely to be followed by a transfer to the ICU. We also found that among patients who use LOE, increased use of interpreters was associated with reduced rates of transfer to the ICU following RRT. Together, these findings suggest improved communication between provider teams and families who use LOE with more consistent interpreter use may help to mitigate disparities in patient outcomes.

## Data Availability Statement

The raw data supporting the conclusions of this article will be made available by the authors, without undue reservation.

## Ethics Statement

The studies involving human participants were reviewed and approved by Seattle Children's Hospital Institutional Review Board. Written informed consent from the participants' legal guardian/next of kin was not required to participate in this study in accordance with the national legislation and the institutional requirements.

## Author Contributions

AO and JM conceptualized and designed the study, carried out analysis, drafted the initial manuscript, and reviewed and revised the manuscript. JRa collected data, carried out analysis, and reviewed and revised the manuscript. TC, SB, JRo, and AA conceptualized and designed the study, provided oversight for the analysis, and reviewed and revised the manuscript. PQ performed statistical analysis, provided feedback regarding study design and data analysis, and reviewed and revised the manuscript. All authors approved the final manuscript as submitted and agree to be accountable for all aspects of the work.

## Funding

This work was funded in part by the Center for Diversity and Health Equity at Seattle Children's Hospital.

## Conflict of Interest

The authors declare that the research was conducted in the absence of any commercial or financial relationships that could be construed as a potential conflict of interest.

## Publisher's Note

All claims expressed in this article are solely those of the authors and do not necessarily represent those of their affiliated organizations, or those of the publisher, the editors and the reviewers. Any product that may be evaluated in this article, or claim that may be made by its manufacturer, is not guaranteed or endorsed by the publisher.
