# Supplementary material for: Association Between Language Use and ICU Transfer and Serious Adverse Events in Hospitalized Pediatric Patients Who Experience Rapid Response Activation
Source: Front Pediatr. 2022 Jul 5;10:872060. doi: 10.3389/fped.2022.872060 (PMC9295993; doi:10.3389/fped.2022.872060)
Supplement: Supplementary file 1 [file Table_1.docx]

Supplemental Table 1. Top 10 diagnoses represented by the study cohort. Data are shown as n (%) out of total 2040.

| **Diagnosis** | **Total patients** | **English-speaking patients** | **Patients who use a language other than English** | **P-value** |
| --- | --- | --- | --- | --- |
| Respiratory distress with or without respiratory failure | 797 (39%) | 689 (39%) | 108 (37%) | 0.144 |
| Congenital cardiac anomaly | 121 (6%) | 104 (6%) | 16 (6%) |  |
| Non-respiratory infection without sepsis | 119 (6%) | 104 (6%) | 15 (5%) |  |
| Gastrointestinal disease | 91 (4%) | 82 (5%) | 9 (3%) |  |
| Acute leukemia or lymphoma | 71 (3%) | 53 (3%) | 18 (6%) |  |
| Solid neoplasm | 65 (3%) | 56 (3%) | 9 (3%) |  |
| Neurologic disease | 75 (4%) | 68 (4%) | 7 (2%) |  |
| Sepsis | 64 (3%) | 51 (3%) | 13 (4%) |  |
| Other congenital anomalies | 40 (2%) | 36 (2%) | 4 (1%) |  |
| Other diagnoses | 597 (29%) | 508 (29%) | 89 (31%) |  |
